# Treatment for Retinopathy of Prematurity in Twins: The Small Twin without High Birth Weight Discordant Is Not at Increased Risk

**DOI:** 10.3390/children9060891

**Published:** 2022-06-15

**Authors:** Enzhong Jin, Zongyi Wang, Lu Yao, Hong Yin, Mingwei Zhao

**Affiliations:** 1 Department of Ophthalmology & Clinical Center of Optometry, Peking University People’s Hospital, Beijing 100044, China; jinenzhong@bjmu.edu.cn (E.J.); wangzongyi929@bjmu.edu.cn (Z.W.); zhaomingwei64@163.com (M.Z.); 2 Eye Diseases and Optometry Institute, Peking University People’s Hospital, Beijing 100044, China; 3 Beijing Key Laboratory of Diagnosis and Therapy of Retinal and Choroid Diseases, Beijing 100044, China; 4 College of Optometry, Peking University Health Science Center, Beijing 100044, China; 5 Department of Ophthalmology, Air Force Medical Center, Air Force Medical University, Beijing 100044, China; yaolufmmu@hotmail.com

**Keywords:** retinopathy of prematurity, twins, risk factors, low birth weight, treatment

## Abstract

Purpose: As common retinopathy is observed in low-birth infants, the characteristics of ROP in twins are worth exploring. The present study tried to demonstrate the risk factors of treatment for retinopathy of prematurity (ROP) in twins both diagnosed with ROP. Methods: A retrospective, institution-based cohort study of 62 premature ROP twin pairs with a mean gestational age (GA) younger than 35 weeks and a birth weight (BW) lower than 2500 g. Only infants with a follow-up period longer than 6 months and complete treatment records were included. The demographic data, treatment requirements and further rescue treatments were all collected and analyzed for all infants according to whether they accepted treatments. Moreover, all twin infants were divided into small and large twin groups according to birth weight, and they were also categorized as three groups according to the treatment requirement including both twins receiving treatment (BT group), one of the twins receiving treatment (ST group) and none of the twins receiving treatment (NT group). Comparisons of demographic data, treatment requirements and further rescue treatments were all conducted according to the different grouping methods. Results: The mean GA of the enrolled infants was (29.29 ± 2.45) weeks with a mean BW of (1335.77 ± 390.36) grams. Among them, 110 infants were mechanically ventilated. Fifty-one of the infants did not receive any treatment and 73 infants received laser or intravitreal injection of anti-VEGF agents. In total, 64 infants only underwent intravitreal injection of anti-VEGF agents or laser treatment, while the other nine infants received scleral buckling or vitrectomy as a necessary treatment when the retinal detachment was observed. No significantly different mechanical ventilation or treatment requirements could be observed between the small twin group and the large twin group (*p* = 0.73, 0.94). The twins in the BT groups showed the lowest BW, while the NT group infants had the highest BW. The GA for the BT, ST and NT groups were (27.86 ± 1.87) weeks, (29.60 ± 1.52) weeks and (31.33 ± 2.39) weeks, respectively, and showed significant differences as well (*p* < 0.001). Conclusion: Being a small twin in twin-paired ROP without a high BW discordant will not increase the risk for treatment requirement or additional surgery necessity with a much more severe stage of ROP.

## 1. Introduction

Retinopathy of prematurity (ROP) is a vasoproliferative disorder of immature retina developed in premature infants with a low birth weight (BW) and a young gestational age (GA) [1]. According to previous studies, lower BW and younger GA are regarded as two of the strongest risk factors for developing ROP and treatment requirements [1,2]. Oxygen supplementation in the early postnatal period is another important risk factor for ROP, but it is also important to prevent a death that can never be discarded [3]. For infants with a lower BW, the immature retinal vessels and higher requirement of oxygen supplementation increase the risk of ROP development, as well as the incidence of the immature, blind preterm baby. In recent years, poor oral health and vitamin D deficiency in pregnant women is also reported to be associated with the prevalence of premature birth and low BW [4]. The analysis of various risk factors helps to improve the understanding of ROP and provides information for subsequent screening, follow-up and treatment.

Being born as twins is also reported to mean an increased risk of developing ROP, though there are conflicting findings regarding the ROP risk in twin or multiple births [5,6,7]. In a recently published study, a higher rate of ROP can be defined in the small twin in preterm discordant twins. [8] However, in this study, all the included twin pairs were with a high BW discordant of ≥20%, which cannot represent the data of twins with or without a diagnosis of ROP. As a special group of individuals, twins with the same gestational age are worthy of further research of intro-group comparison and population analysis for the occurrence risk and treatment requirement of ROP.

According to our previous studies and data from other countries, we found a phenomenon that the infants who suffered severe ROP were larger than those in developed countries, and with a much broader range of BWs and GAs than that in industrialized countries [9,10,11]. In this case, the screening criteria in China were deliberately set to include broad ranges of ages and weights (34 weeks GA or 2000 g BW) [12]. Based on the national difference in the ROP epidemiological situation, ROP twins no larger than 35 weeks were all included in the present study. In the present study, we tried to collect and analyze the background and treatment characteristics of ROP in twins. The twins were divided into the large ROP group and small ROP group, and the risk of developing much more severe ROP and requiring more treatment were both compared. A different point of view from the previous study can be obtained in our present study, which can provide valuable information for the recognition and management of ROP, especially for twins with ROP.

## 2. Methods

### 2.1. Study Design

This is a retrospective, institution-based cohort study of ROP twins attending a single Children’s Eye Center between January 2017 and December 2019 approved by the Ethical Review Committee of Peking University People’s Hospital (Beijing, China), which was conducted in accordance with the Declaration of Helsinki (2017PHB179-01). Written informed consent was obtained from the parents of each twin pair before receiving any kinds of treatments as necessary.

The examination of binocular indirect ophthalmoscopy was performed for all included ROP twins by the same skilled retinal specialist (H.Y.) and the whole medical records were carefully reviewed. The fundus examinations were performed with indirect ophthalmoscope after pupil dilation for all ROP infants. The history of pregnancy and delivery, oxygen inhalation and treatment records were collected and further analyzed.

First, the analyses for the whole twin infants were performed in our present study. For all included infants, only data for one eye of each infant were collected for analysis and comparison. Second, all the included twins were divided into two groups according to the weight of infants for each twin pair, the heaver one in a twin pair was included in the large twin group and the lighter one was included in the small twin group. After the grouping, the comparisons between large twin group and small twin group were performed. Third, the ROP twins were categorized into three groups according to the treatment requirement including both twins receiving treatment (BT group), one single twin receiving treatment (ST group) and none of the twins receiving treatment (NT group) for further analysis.

### 2.2. Patients

All included infants were born as preterm twins and both diagnosed as ROP with GA younger than 35 weeks, and BW lower than 2500 g. Both twins of each twin pair were born on the same day. The patients who did not attend for re-exam or with a follow-up period shorter than 6 months were excluded. The diagnosis of ROP was according to the international classification for ROP (ICROP) [13]. According to previous study, a birthweight difference higher than 20% was defined as high discordant [8]. In our study, the twins were also divided into three subgroups according to the BW discordant. The intertwin BW discordance of <5%, 5–9% and 10–20% were defined as different subgroups for further analysis.

Three groups of twins were enrolled with criteria as follows: (1) BT group twins both receiving treatments including anti-VEGF, laser, pars plana vitrectomy (PPV) and scleral buckling surgery (SBS); (2) ST group twins with one of the twins receiving any treatments; (3) NT group twins neither receiving any treatment.

### 2.3. Treatment

All the included infants undergoing treatments fulfilled the criteria of treatment in accordance with the guidelines of the Early Treatment for Retinopathy of Prematurity Cooperative Group [14]. For the eyes with threshold or pre-threshold Type I ROP, intravitreal injections of anti-VEGF agents or laser were applied. The eyes without regression of plus disease or ridge with one injection would receive a repeat injection during the follow-up period, as well as those eyes with a recurrence of ROP after regression. PPV or SBS were performed only if it is necessary when retinal detachment was observed.

The dosage of anti-VEGF agents including conbercept and ranibizumab for the ROP infants was reduced to 0.25 mg/0.025 mL as half of the adult dose for other retinal vascular diseases according to our previous study [15]. The procedure of intravitreal injection was performed by a skilled retina specialist (H.Y.) according to the guideline of an expert panel [16]. The location of injections was in the temporal quadrant and 1.0–1.5 mm posterior to the limbus. Laser, PPV and SBS surgeries were also performed by the same skilled retina specialist (H.Y.).

### 2.4. Statistical Analysis

Statistical analyses were performed using Statistical Package for the Social Sciences for Windows software, version 24.0 (SPSS, Inc., Chicago, IL, USA). Continuous variables were presented as mean ± standard deviation, and median (P25, P75). To compare the characteristics of all infants receiving different treatments, one-way analysis of variance (ANOVA), Kruskal–Wallis test and Chi-square test were used. The differences in the characteristics and treatments of infants grouped according to small and large twins and according to whether both twins received treatment were assessed with one-way ANOVA, Kruskal–Wallis test, and Chi-square test. Bonferroni test and Dunn’s test were used for post-hoc multiple comparisons. All *p*-values were two-sided and considered statistically significant when less than 0.05.

## 3. Results

One hundred and twenty-four ROP infants, namely 62 twins with or without having had treatments were finally included in this retrospective cohort study. Among them, mechanical ventilation was performed in 90% of infants, 51/124 (41.1%) of the included infants were female while 73/124 (58.9%) were male. The mean GA of all infants was (29.29 ± 2.45) weeks with a mean BW of (1335.77 ± 390.36) grams. Fifty-one of the infants did not receive any treatment and the other 73 infants received laser or anti-VEGF treatment. Seven twin pairs were excluded from our study because of two reasons. Five of them did not attend a follow-up, and the other two did not attend a follow-up within 6 months (Figure S1).

Among the 73 infants receiving treatment, 64 infants only underwent intravitreal injection of anti-VEGF agents or laser treatment, while the other nine infants received scleral buckling or vitrectomy which is a necessary treatment when the retinal detachment was observed during the follow-up period. Among the nine infants requiring scleral buckling or vitrectomy, four infants underwent vitrectomy while five infants underwent scleral buckling. All infants achieved complete retinal reattachment except two infants with stage 5 ROP, who showed partial retinal reattachment. The infants who underwent any treatment showed lower BW and younger GA than those who did not require treatment (*p* < 0.001), and a higher percentage of mechanical ventilation percentage can also be observed in infants who underwent any treatment (*p* = 0.003). No gender difference can be found between the infants with or without treatment (*p* = 0.52) (Table 1).

The demographic characteristics and treatments of infants grouped according to the small or large twin groups were analyzed. The mean BW discordance of all included twins was (12.88 ± 13.67) % with a median of 8.42%. No significant difference in mechanical ventilation or treatment requirement can be observed between the small twin group and the large twin group (*p* = 0.73, 0.94) (Table 2).

Subgroup analysis was also performed for the twin groups according to different BW discordance, including discordance of <5%, 5–9% and 10–20% groups. For the subgroup analysis, no significant difference of treatment requirement can be observed between the small twin group and the large twin group for different BW discordance groups (*p* = 0.41, 0.52, 0.55) (Tables S1–S3).

When all the twins were categorized into BT, ST and NT groups according to the treatments they underwent, the BW, GA, BW discordance and oxygen inhalation were all compared among the three groups. A significant difference in BW can be observed for BT, ST and NT groups as (1139.88 ± 284.01) g, (1315.90 ± 285.46) g and (1667.92 ± 399.04) g, respectively (*p* < 0.001). The twins in the BT groups showed the lowest BW, while the NT group infants had the highest BW. The GA for the BT, ST and NT groups were (27.86 ± 1.87) weeks, (29.60 ± 1.52) weeks and (31.33 ± 2.39) weeks, respectively, and showed a significant difference as well (*p* < 0.001). The median BW discordance for the three groups was 8.6%, 11.5% and 7.3%, with no significant difference. The NT group infants showed the lowest percentage in mechanical ventilation percentage (72.2%) while 100.0% of infants in the ST group and 98.2% of the BT group received mechanical ventilation when born (Table 3).

Focusing on the treatments of the ROP twins, the ST and BT groups were included in the analysis. For the ST group, 12/15 (80.0%) infants only received anti-VEGF agents or laser treatment while 3/15 (20.0%) infants required scleral buckling or vitrectomy. For the BT group, 52/58 (89.7%) infants underwent anti-VEGF treatment while 6/58 (10.3%) infants received scleral buckling or vitrectomy treatment. No significant difference in treatment can be observed between the two groups (*p* = 0.38) (Table 4).

Last but not least, no complication such as intravitreal hemorrhage, endophthalmitis, glaucoma or cataract was observed for all the included infants.

## 4. Discussion

The purpose of our present study is to discuss whether smaller BW is associated with the requirement of treatment and additional treatment in the population of ROP infants born as twins. Unlike the previous studies, all the included twin infants were diagnosed as ROP, and the comparisons were performed among the ROP infants but not just premature twins [8]. We also report the BW, GA and oxygen inhalation differences between twins who underwent different treatments. To the best of our knowledge, this is the first retrospective cohort study that categorized the ROP twins into different groups according to their treatment choices. Additionally, it is the first study showing no difference between a smaller BW and a larger BW for treatment requirements in unselected ROP twins.

As we all know, low BW and young GA are the two strongest risk factors for developing ROP as well as excessive oxygen supply [1]. Multiple gestations are associated with increased risk for preterm birth and smaller BW, which may affect the risk of ROP [17]. It is also well known that low BW rate and preterm delivery rate are much higher in twins than in singletons [18]. Since ROP is much more common in twins than in singletons, it is worthwhile investigating the risk factors of treatment requirements in ROP twins and whether more treatments were required for certain groups of ROP twins.

The major findings of the current study included: First, BW, GA and oxygen inhalation were risk factors for the infant needing treatment among the population of ROP twins. Second, for the ROP twins with a mean BW discordance of (12.88 ± 13.67)%, being the small twin is not a risk factor for requiring treatment, which means the larger one and smaller one in ROP twins share a similar risk of treatment requirement once both of them were diagnosed with ROP with low BW discordance. Third, when the ROP twins were categorized into three groups including both twins receiving treatment (BT group), one of the twins receiving treatment (ST group) and none of the twins receiving treatment (NT group), the BT group of infants showed lower BW and younger GA. On the other hand, the proportion of BW discordance showed no differences between the three groups.

In our present study, the ROP infants receiving scleral buckling or vitrectomy showed even older GA than infants undergoing laser or anti-VEGF treatment. As we all know, infants with a younger GA develop severe ROP much easier. Apart from the infants that required further treatment with the more severe ROP in our present study, younger GA and lower BW were not observed. This may be because most infants undergoing scleral buckling or vitrectomy in our study are stage 4A ROP cases, and 7/9 (77.78%) of them were first treated with intravitreal injection of anti-VEGF agents. Such a high proportion of infants not requiring surgery at the first visit meant a much less severe ROP stage for these nine infants. On the other hand, this may also be associated with the small scale of this group.

In previous studies, whether increased risk could be defined in multiple gestations compared with a single birth infant is still inconsistent. In the CRYO-ROP study, the multiple-birth infants were found to have an increased risk of treatment-requiring ROP than singleton infants [2]. However, different or even opposing viewpoints were also reported in some other studies showing no differences between singleton and multiple births or even higher incidence of ROP in singletons [6,19,20]. In our present series, all the infants were diagnosed as ROP with a mean GA of (29.29 ± 2.45) weeks, showing a group of ROP infants in a premature twin population, but no further conclusion between singleton and multiple births can be made without a control group.

In the present study, no differences in primary and rescue treatment requirements can be observed between the small twin group and the large twin group, which provides a novel point for the twin-paired ROP that no additional treatment was required for the small twin in the twin-paired ROP infants. However, as we all know, low BW was an identified risk factor for ROP [1,2]. In a previous respective cohort, 45 preterm twin pairs born with a BW discordance of ≥20% (mean BW discordance of 35%) were analyzed and the rate of ROP was higher among the small twins, and low BW was defined as the main factor contributing to the development of ROP [8]. The discrepancy between our cohort and this previous cohort may due to the difference in BW discordant. The median of the BW discordance for our study was no more than 11.5%, which meant that a small difference in BW between the twin pairs could explain the similarity of treatment between big and small twins. Similar to our study, a co-twin study also showed no significant difference in the rate of ROP between large and small twins with a BW discordance of less than 15% [21]. Further studies with a larger scale including more twin-paired ROP with a much wider range of discordance are required for a more consistent result. On the other hand, our outcome may also provide indirect evidence for the importance of GA for the ROP in twins because the twins with the same GA finally underwent the same treatments.

When the twin-paired ROP were categorized into BT, ST and NT groups, both lower BW and younger GA were found to be associated especially with the BT group, which suggested BW and GA were both risk factors for more treatment requirements in ROP twins. Such kinds of categories showed a novel perspective for identifying the treatment choice for different twins diagnosed with ROP. For the ROP twins with a lower BW and younger GA, a more serious stage of ROP might be diagnosed and more treatment might be required. Among all infants who underwent treatment, only one of them did not receive mechanical ventilation after birth with a GA of 28 weeks and BW of 1600 g. The bigger twin in the twin pair was 150 g heavier than his brother but still had a high risk of needing ROP treatment [22]. Both of them only received one injection and did not require further rescue treatment. Generally, almost all of the ROP twins requiring treatment received mechanical ventilation after birth. This result observed in our present study also suggested that oxygen inhalation in the treatment of premature infants may play an important role in the development of ROP. A further intra-group comparison was also performed. The characteristics of the treated and untreated infants were compared in the ST group, but no significant difference in BW, gender or the oxygen inhalation situation could be demonstrated between them.

As a retrospective cohort, our study has several limitations. First, it was not prospectively designed and infants included in the present study were ROP patients attending a single Children’s Eye Center. The study is not generalizable to the overall population of twins, but of course, it can help us for further analysis of the twins for the development and treatment of ROP. Second, the scale of the present study was small for demonstrating the characteristics of ROP twins, though it was relatively bigger than the previous studies [6], which means the evidence is more convincing. Third, long-term follow-up of the structural and functional outcomes for ROP twins was not analyzed in this study, which could better improve our understanding of ROP twins. Since this was a retrospective cohort study, a longer follow-up time is still required in the future with a better future design. The strengths of this study should also be mentioned. As a study focused on the primary treatment and rescue treatment requirements for twin-paired ROP, it is much different from the previous descriptive studies which pay most attention to the development and severity of ROP in premature twins. As a single-center study, the same standards for inclusion criteria, diagnosis of ROP, treatment choice and performance, and management protocol were applied, which made this study more reliable. Moreover, the ROP twins were categorized into three groups receiving different treatments, and our results suggested the ROP twins with a lower BW may require more treatment but not more rescue treatment. This novel category provided a special viewpoint for the treatment analysis of twin-paired ROP infants.

In conclusion, the data of our present study demonstrated the development and treatment characteristics of twins that are both diagnosed with ROP. The BW discordance was found to be an important factor affecting the treatment requirement of ROP twins. Although the overall incidence of ROP is thought to be higher in twin birth infants with relatively lower birth BW, small twins without a high BW discordant are not at increased risk for requiring treatment or additional rescue treatment although they are diagnosed with a more severe stage of ROP. High concentration oxygen supplements are another crucial aspect of ROP incidence and development and therefore require more attention. Additional large-scale prospective research is required for further determination of the risk factors and more precise management of ROP in twins.

## 5. Conclusions

In summary, the present study focused on the severity and treatment requirement of ROP in a twin cohort. No increasing risk of more treatments for the small twin in a twin pair with low BW discordance (<15%) can be identified, which suggested that the BW discordance may be an important risk factor of treatment requirement in ROP twins only if it is big enough. The relationship between the BW discordance and severity of ROP may be associated with the low BW itself and hypoplasia of the retinal vasculature.

## Figures and Tables

**Table 1:** Demographic characteristics and treatments of all ROP infants.

Variable	Total	Non-Therapy	Laser/Intravitreal Injection of Anti-VEGF	Scleral Buckling/Vitrectomy	*p*
n	124	51	64	9	-
Birth weight, g (Mean ± SD)	1335.77 ± 390.36	1561.76 ± 395.30	1167.27 ± 306.24	1253.33 ± 259.48	<0.001
Gestational age, week (Mean ± SD)	29.29 ± 2.45	30.82 ± 2.30	28.08 ± 1.92	29.22 ± 1.79	<0.001
Gender					
Female, % (N)	41.1% (51)	47.1% (24)	37.5% (24)	33.3% (3)	0.52
Male, % (N)	58.9% (73)	52.9% (27)	62.5% (40)	66.7% (6)	
Oxygen inhalation					
None, % (N)	9.1% (11)	19.6% (10)	1.6% (1)	0.0% (0)	0.003
Mechanical ventilation, % (N)	90.0% (110)	80.4% (41)	98.4% (60)	100.0% (9)	

**Table 2:** Characteristics and treatments of infants grouped according to small or large twins.

	Small Twin	Large Twin	*p*
Birth weight, g (Mean ± SD)	1257.98 ± 354.00	1413.55 ± 411.88	0.03
Oxygen inhalation			
None, % (N)	9.5% (6)	8.6% (5)	0.73
Mechanical ventilation, % (N)	90.5% (51)	91.4% (53)	
Treatment			
None, % (N)	41.9% (26)	40.3% (25)	0.94
Laser/ intravitreal injection of anti-VEGF, % (N)	51.6% (32)	51.6% (32)	
Scleral buckling/vitrectomy, % (N)	6.5% (4)	8.1% (5)	

**Table 3:** Characteristics of infants grouped according to whether both twins received treatment.

	BT Group	ST Group	NT Group	*p*
n	58	30	36	-
Birth weight, g (Mean ± SD)	1139.88 ± 284.01	1315.90 ± 285.46	1667.92 ± 399.04	<0.001
Gestational age, week (Mean ± SD)	27.86 ± 1.87	29.60 ± 1.52	31.33 ± 2.39	<0.001
BW discordance, % (Md, P25, P75)	8.6 (4.3, 12.2)	11.5 (4.7, 23.5)	7.3 (3.2, 16.0)	0.30
Oxygen inhalation				
None, % (N)	1.8% (1)	0.0% (0)	27.8% (10)	<0.001
Mechanical ventilation, % (N)	98.2% (54)	100.0% (30)	72.2% (26)	

BT: Both twins received treatment; ST: Single twin received treatment and the other did not require treatment; NT: None of the twins received treatment.

**Table 4:** Treatments of infants grouped according to whether both twins received treatment.

Treatment	ST Group	BT Group	*p*
Laser/ intravitreal injection of anti-VEGF agents, % (N)	80.0% (12)	89.7% (52)	0.38
Scleral buckling/vitrectomy, % (N)	20.0% (3)	10.3% (6)	

ST: Single twin received treatment and the other did not require treatment; BT: Both twins received treatment.

## Data Availability

The data and material used to support the findings of this study are available from the corresponding author upon request.

## Supplementary Materials

The following supporting information can be downloaded at: https://www.mdpi.com/article/10.3390/children9060891/s1. Figure S1: Flowchart for the inclusion of twins. Table S1: Characteristics and treatments of infants grouped according to small or large twins (BW discordance <5%). Table S2: Characteristics and treatments of infants grouped according to small or large twins (BW discordance 5~9%). Table S3: Characteristics and treatments of infants grouped according to small or large twins (BW discordance 10~19%).

## Abbreviations

ROP: Retinopathy of prematurity; BW: Birth weight; GA: Gestational age; IVR: Intravitreal ranibizumab; PMA: Postmenstrual age; VEGF: Vascular endothelial growth factor; BT: Both twins receiving treatment; ST: One of the twins receiving treatment; NT: None of the twins receiving treatment (NT group).
