# Supplementary material for: Treatment for Retinopathy of Prematurity in Twins: The Small Twin without High Birth Weight Discordant Is Not at Increased Risk
Source: Children (Basel). 2022 Jun 15;9(6):891. doi: 10.3390/children9060891 (PMC9222126; doi:10.3390/children9060891)
Supplement: Supplementary file 1 [file children-09-00891-s001.zip › children-1737668-SI.pdf]

Supplementary materials

Table S1. Characteristics and treatments of infants grouped according to small or large twins (BW discordance <5%)

	Small twin	Large twin	P
Birth weight, g (Mean±SD)	1266.58±381.76	1300.00±394.21	0.51
Oxygen inhalation			
None, % (N)	11.1% (2)	11.1% (2)	1.00
Mechanical ventilation, % (N)	88.9% (16)	88.9% (16)	
Therapy			
None, % (N)	47.4% (9)	31.6% (6)	0.41
Laser/ intravitreal injection of anti-VEGF, % (N)	52.6% (10)	63.2% (12)	
Scleral buckling/vitrectomy, % (N)	0% (0)	5.3% (1)	

Table S2. Characteristics and treatments of infants grouped according to small or large twins (BW discordance 5~9%)

	Small twin	Large twin	P
Birth weight, g (Mean±SD)	1396.00±380.41	1493.87±405.94	0.50
Oxygen inhalation			
None, % (N)	7.1% (1)	0.0% (0)	0.48
Mechanical ventilation, % (N)	92.9% (13)	100.0% (15)	
Therapy			
None, % (N)	40.0% (6)	53.3% (8)	0.52
Laser/ intravitreal injection of anti-VEGF, % (N)	53.3% (8)	33.3% (5)	
Scleral buckling/vitrectomy, % (N)	6.7% (1)	13.3% (2)	

Table S3. Characteristics and treatments of infants grouped according to small or large twins (BW discordance 10~19%)

	Small twin	Large twin	P
Birth weight, g (Mean±SD)	1224.38±245.44	1392.00±297.34	0.09
Oxygen inhalation			
None, % (N)	12.5% (2)	12.5% (2)	1.00
Mechanical ventilation, % (N)	7.5% (14)	7.5% (14)	
Therapy			
None, % (N)	43.8% (7)	31.3% (5)	0.55
Laser/ intravitreal injection of anti-VEGF, % (N)	43.8% (7)	62.5% (10)	
Scleral buckling/vitrectomy, % (N)	12.5% (2)	6.3% (1)	

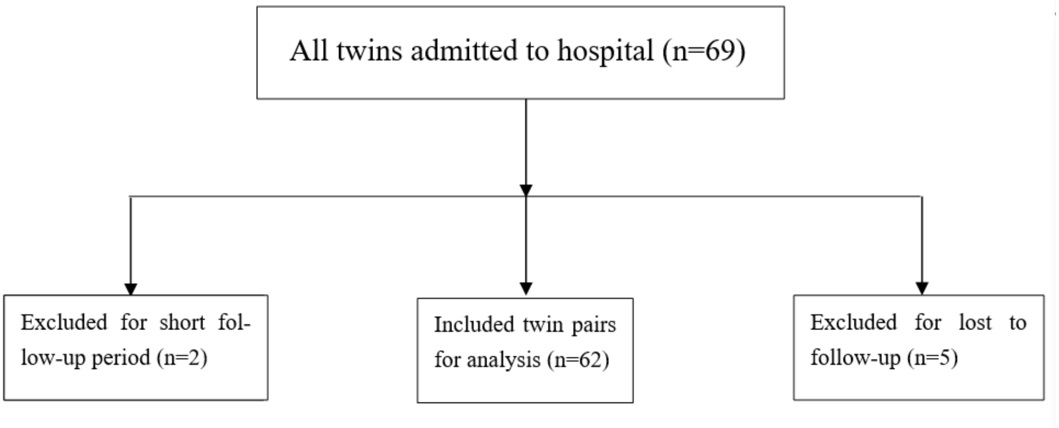

Figure S1. Flowchart for the inclusion of twins
